# GLP-1 receptor agonists for weight reduction in people living with obesity but without diabetes: a living benefit–harm modelling study

**DOI:** 10.1016/j.eclinm.2024.102661

**Published:** 2024-05-27

**Authors:** Hannah Moll, Eliane Frey, Philipp Gerber, Bettina Geidl, Marco Kaufmann, Julia Braun, Felix Beuschlein, Milo A. Puhan, Henock G. Yebyo

**Affiliations:** a Department of Epidemiology, Epidemiology, Biostatistics, and Prevention Institute, University of Zurich, Zurich, Switzerland; b Department of Chemistry and Applied Biosciences, Institute of Pharmaceutical Sciences, ETH, Zurich, Switzerland; c Department of Endocrinology, Diabetology and Clinical Nutrition, University Hospital of Zurich and University of Zurich, Zurich, Switzerland; d Medizinische Klinik und Poliklinik IV, Klinikum der Universität, Ludwig-Maximilians-Universität, Munich, Germany; e The LOOP Zurich - Medical Research Center, Zurich, Switzerland

**Keywords:** Obesity, Metabolic disease, GLP-1 RA, Weight loss, Weight management, Benefit-harm assessment

## Abstract

**Background:**

The benefit of Glucagon-like Peptide-1 (GLP-1) receptor agonists (RAs) in weight reduction against potential harms remains unclear. This study aimed at evaluating the benefit-harm balance of initiating GLP-1 RAs versus placebo for weight loss in people living with overweight and obesity but without diabetes.

**Methods:**

We performed benefit-harm balance modelling, which will be updated as new evidence emerges. We searched for randomised controlled trials (RCTs) in PubMed, controlled trials registry, drug approval and regulatory documents, and outcome preference weights as of April 10, 2024. We synthesize data using pairwise meta-analysis to estimate the effect of GLP-1 RAs to inform the benefit-harm balance modelling. We predicted the absolute effects of the positive and negative outcomes over 1 and 2 years of treatment using exponential models. We applied preference weights to the outcomes, ranging from 0 for least concerning to 1.0 for most concerning. We then calculated whether the benefit of achieving 5% and 10% weight loss outweighed the harms on a common scale. The analyses accounted for the statistical uncertainties of treatment effects, preference weights, and outcome risks.

**Findings:**

We included 8 RCTs involving 8847 participants. The pooled average age was 46.7 years, with the majority being women (74%) and people living with obesity (96%). Of 1000 persons treated with GLP-1 RAs for 2 years, 375 (95% confidence interval 352 to 399) achieved a 10% weight loss, and 318 (296 to 339) achieved a 5% weight loss compared to those treated with placebo. Several harm outcomes were more frequent in the GLP-1 RA group, including 41 abdominal pain events per 1000 persons over 2 years (19 to 69), cholelithiasis (8, 1 to 21), constipation (118, 78 to 164), diarrhoea (100, 42 to 173), alopecia (57, 10 to 176), hypoglycaemia (17, 1 to 68), injection site reactions (4, −3 to 19), and vomiting (110, 80 to 145) among others. Achieving a 10% weight loss with GLP-1 RA therapy outweighed the cumulative harms, with a net benefit probability of 0.97 at year 1 and 0.91 at year 2. The absolute net benefit was equivalent to 104 (100 to 112) per 1000 persons achieving a 10% weight loss over 2 years without experiencing any worrisome harm. A 5% weight loss did not show a net benefit, with probabilities of 0.13 and 0.01 at year 1 and year 2, respectively. However, these benefits were sensitive to preference weights, suggesting that even a 5% weight loss could be net beneficial for individuals with less concern about harm outcomes. The net benefit for a 10% weight loss was highest for semaglutide, followed by liraglutide and tirzepatide, with 2-year probabilities of 0.96, 0.72, and 0.60, respectively.

**Interpretation:**

The benefit of GLP-1 RAs exceeded the harms for weight loss in the first 2 years of treatment, yet the net benefit was dependent on individual’ treatment goals (10% or 5% weight loss) and willingness to accept harms in pursuit of weight loss. This implies that treatment decisions have to be personalized to individuals to optimize benefits and reduce harms and overuse of treatments. Due to varying evidence, especially regarding harm outcomes across studies, it is necessary to continuously update and monitor the benefit-harm balance of GLP-1 RAs.

**Funding:**

10.13039/501100001711SNSF and LOOP Zurich.

**Research in context**

**Evidence before this study**

We searched PubMed as of April 10, 2024, for any benefit-harm (risk) assessments (and related studies) on GLP-1 RAs. We found no relevant studies except systematic studies on individual benefit and harm outcomes in isolation. The systematic reviews showed that GLP-1 RAs effectively reduce weight in people living with obesity (but without diabetes), particularly in the initial years of treatment. However, they also reported several associated harm outcomes that could deter the initiation of GLP-1 RA therapy for some individuals. Without systematic and quantitative assessments, the balance of benefit in terms of weight reduction against the potential harms would be unclear.

**Added value of this study**

The current study evaluated the benefit-harm balance, factoring in all negative and positive outcomes on the same scale. It determined whether GLP-1 RAs provide more good than harm (net benefit) and, if so, which individuals derive the net benefit. The findings showed that the 10% weight loss outweighed the harms over 2 years of treatment with GLP-1 RAs, but the 5% weight loss did not. However, this net benefit was highly dependent on outcome preferences. For some risk-accepting individuals, even the 5% weight loss was found to be net beneficial.

**Implications of all the available evidence**

Our study demonstrated important findings on how the overall net benefit of GLP-1 RAs should be assessed. The study showed that GLP-1 RAs offer a net benefit for some individuals but not for others, depending on weight loss target (10% or 5%) and their willingness to accept or avoid harms. The net benefit was sensitive to patient preferences, implying that the treatment should be personalized to individuals, taking into account patient treatment goals, outcome risks, and preferences. The evidence on GLP-1 RAs is rapidly evolving. It is important to continuously monitor and assess the long-term treatment efficacy and safety and the benefit-harm balance.

## Introduction

Glucagon-like peptide-1 receptor agonists (GLP-1 RAs) were initially approved for improvement of metabolic control in patients living with diabetes.^1^ However, recent randomised controlled trials (RCTs) have demonstrated the efficacy of some GLP-1 RAs for weight reduction in adults even without diabetes, leading to semaglutide and liraglutide approval as adjuncts to a calorie-restricted diet and exercise for weight management.^2^, ^3^, ^4^ Tirzepatide, a newer dual receptor agonist, has also been demonstrated to induce greater weight reductions,^5^ and has recently been approved for patients without diabetes in some countries.^6^ Early-phase investigations on other emerging medications, particularly retatrutide and orforglipron that target multiple incretin hormone pathways, have shown even greater effects on weight loss.^7^^,^^8^

GLP-1 RAs modulate appetite regulation, which includes slowing gastric emptying, increasing satiety, and reducing appetite, resulting in weight loss.^9^^,^^10^ The benefits of weight reduction are consistent across RCTs, with great potential to address the obesity pandemic. Although less studied, GLP-1 RAs may also contribute to a reduction in the risk of weight-related comorbidities.^11^^,^^12^ The SELECT trial indicated a 20% reduction in recurrent cardiovascular disease (CVD) events with the GLP-1 RA analogue semaglutide.^13^ Despite these impressive clinical effects, questions remain regarding whether the benefits of weight reduction outweigh the associated harms of GLP-1 RAs, including their cost-effectiveness and economic impact. Another challenge is the rapid and steep increase in off-label GLP-1 RAs prescriptions, often lacking physicians’ supervision and posing a relevant risk of overuse and misuse.^14^ The widespread usage is largely exacerbated by social media hype and celebrity endorsements for “cosmetic” weight loss.^15^ Extensive use—without scrutinising in whom the benefits outweigh the harms—not only escalates healthcare expenditure but, more critically, has led to supply shortages, restricting access for individuals living with diabetes.^16^, ^17^, ^18^, ^19^

The use of GLP-1 RAs for weight control in patients living without diabetes is a relatively new indication for weight management in people living with obesity, with largely unknown long-term effects. However, as evidence on the topic is accumulating rapidly, there is a need for continuous evaluation and monitoring of the benefit-harm balance to guide up-to-date decisions. In the present study, we aim to conduct a benefit harm-balance assessment of GLP-1 RAs, which will be regularly updated as evidence emerges to inform decision-makers, including regulatory bodies, clinical guideline developers, and personalized treatment planning.

## Methods

### Study design and participants

We performed a benefit-harm balance modelling, which will be updated as new evidence emerges. We performed a literature search and synthesized data using pairwise meta-analysis to estimate the effect of GLP-1 RAs on the various outcomes to inform the benefit-harm balance modelling. Our target population was people aged 18 years or older without diabetes, with body mass index (BMI) of ≥30 kg/m^2^ or ≥27 kg/m^2^ in the presence of at least one weight-related comorbidity, including hypertension, dyslipidemia, obstructive sleep apnoea, or CVD.

### Treatments

We included approved GLP-1 RAs for people without diabetes. We compared 3 mg daily liraglutide, 2.4 mg weekly semaglutide, and a combined dose of 10 mg and 15 mg weekly tirzepatide with placebo, all administered subcutaneously. Of note, although tirzepatide is additionally a glucose-dependent insulinotropic polypeptide (GIP) RA,^20^ in this study we refer to all treatments as GLP-1 RAs for better readability.

We included RCTs where participants received add-on lifestyle counselling to maintain a deficit of 500 calories per day based on their individual energy requirements and to be physically active for at least 150 min per week in both the treatment and placebo arms.

### Outcomes

The benefit outcome was achieving a weight loss of ≥5% or ≥10% from baseline, each considered as separate end points in the analysis. Since there were too many harms (adverse effects) reported across RCTs, we selected harms that were frequent (≥1%), as labelled by the Swiss drug compendium, or any serious harms regardless of their frequency.^21^, ^22^, ^23^ Accordingly, we included abdominal pain, constipation, diarrhoea, dizziness, dyspepsia, eructation, fatigue, flatulence, headache, hypoglycemia, injection site reactions, nausea, and vomiting as frequent harms, as well as alopecia, cholecystitis, cholelithiasis, and pancreatitis as moderate to serious harm outcomes. Furthermore, in a separate analysis, we considered treatment discontinuation due to adverse effects as the only negative outcome, disregarding the specific harm outcomes listed above.

### Data sources

The input parameters for the benefit-harm balance modelling were the relative treatment effects of GLP-1 RAs as compared to placebo, the benefit and harm outcome incidences in untreated individuals, and preference weights (relative importance) of the outcomes.^24^, ^25^, ^26^, ^27^

A careful selection of valid and applicable evidence relevant to the given decision context is a key step in a benefit-harm analysis.^28^, ^29^, ^30^ The relative treatment effect and baseline risks were obtained from RCTs. Three members (HM, EF, HGY) independently conducted a PubMed search for published systematic reviews on GLP-1 RAs as of April 10, 2024. We found 20 systematic reviews from which we retrieved RCTs evaluating the effects of GLP-1 RAs on people living without diabetes. To avoid missing any recently published studies, a separate PubMed search was performed for RCTs that may be published later than the last systematic review. We also searched for drug approval documents, RCT registry, and conference papers. As a living benefit-harm balance evaluation, the evidence will be updated continuously as new evidence emerges. We extracted the required data and employed a random-effects meta-analysis to estimate the relative effects, as well as pooled outcome incidences.

However, since empirical preference weights specific to our population for the outcomes were not available, we considered a range of generic weights for mild, moderate, and severe outcomes commonly used in other studies.^26^^,^^31^^,^^32^ A preference weight measures “*how worrisome a health condition is to individuals*”. It helps to indicateing the patient’s willingness to accept or avoid risks in the analysis. For mild events like dizziness, fatigue, headache, hypoglycemia, and injection site reactions, and gastrointestinal harm outcomes, we assigned a preference weight of 0.10, with 0 signifying no worrisome harm at all and 1.00 signifying the most worrisome outcome. To avoid the risk of double counting and overestimation of harms, we halved the weights to 0.05 specifically for the gastrointestinal-related harm outcomes (such as abdominal pain, constipation, diarrhoea, dyspepsia, eructation, flatulence, nausea, upper abdominal pain, and vomiting) that may be correlated. We assigned 0.25 for the relatively more serious outcomes, including alopecia, cholecystitis, cholelithiasis, and pancreatitis. The benefit of achieving a weight loss target of ≥5% and ≥10% received weights of 0.1 and 0.25, respectively.

We deducted these preference weights by anchoring with outcomes from other studies, such as death as the most serious outcome with a weight of 1.00, myocardial infarction with 0.42 to 0.65, and mild to moderate diarrhoea with 0.13.^32^^,^^33^ Since a single value would not capture the real-world variation, we accounted for uncertainty values of 0–0.10 for the preference weight of 0.05, 0.05–0.15 for 0.10, and 0.20–0.30 for 0.25, with a uniform distribution.

To assess whether empirical aggregate preferences are necessary or if individual patient preferences should be used in decision-making, we additionally conducted the analysis with wider ranges of preference values for selected outcomes (see sensitivity analysis).

### Statistical analysis

The benefit-harm balance modelling compared the cumulative benefit events versus cumulative harm events over 1 and 2 years, time horizons equivalent to most follow-up periods of the available RCT. Our analyses focused on the treatment initiation of GLP-1 RAs for weight loss, and not on the treatment use for weight stabilization. Cumulative events of positive and negative outcomes were predicted using exponential models for GLP-1 RA-treated and untreated groups assuming constant outcome ratets over the time horizons. The difference in predicted outcomes between the two treatment groups (absolute effect) was calculated, adjusted by preference weights, and summed across all outcomes to obtain the benefit-harm index (or net benefit). The net benefit index had either negative value (harm outweighs benefit), positive value (benefit outweighs harm) or zero (equipoise).

We performed this analysis repeatedly 100000 times considering the statistical uncertainty of the relative effect estimates, incidences of the benefit and harm outcomes in the untreated individuals, and preferences to generate a distribution of the benefit-harm balance indices. Since the benefit-harm index was a product of several parameters, its interpretation can be complex. To simplify interpretation, we transformed the index into equivalent event of achieving a 10% weight loss per 1000 persons over 2-years. This transformed metric, as an indicator for net benefit, can be interpreted as the number of people achieving the 10% weight loss, without experiencing any worrisome harms. The analyses included a bootstrapping method with 1000 samples to estimate 95% uncertainty intervals based on the 2.5^th^ and 97.5^th^ centiles in the distributions of absolute effects and net benefit.

We additionally calculated the probability that individuals obtained a net benefit from GLP-1 RAs from the distribution of the benefit-harm index. The model is described elsewhere.^24^, ^25^, ^26^, ^27^ See additional explanation in Appendix pp1. The GLP-1 RAs were considered to be net beneficial when the probability of net health benefit reached at least 0.60. We chose 0.60, instead of 0.50, to ensure a certain benefit since the net benefit at a probability of 0.5 would be zero. This is because most people are slightly risk-averse and they would not take the treatments if they could not obtain any benefit.

### Sensitivity analysis

We conducted different sensitivity analyses to examine the robustness of the analysis. Firstly, guided by insights from some of the RCTs,^34^, ^35^, ^36^, ^37^, ^38^, ^39^ we updated our model with the assumption that some of the harm rates do not remain constant over the time horizons. Some of them could be more frequent during the initial treatment, and subsequently decline due to the tolerance and attenuation of the placebo effect. As such, we partitioned the time horizons into small parts. We assumed that some outcome rates would remain similar during the first three months of treatment initiation, followed by a gradual decrease over the following consecutive 3-month periods until the end of the first year, with decline rates by 10%, 40%, and 50%, respectively. After the first year, the incidence rates were assumed to remain constant. This assumption was applied to the common harm outcomes, such as abdominal pain, constipation, diarrhoea, dizziness, dyspepsia, eructation, fatigue, flatulence, headache, nausea, and vomiting. Secondly, we broke down the analysis by drug type. Thirdly, we assessed the impact of varying preferences on the net benefit for selected outcomes. Finally, we replaced the specific harm outcomes with treatment discontinuation due to adverse effects to assess the net benefit.

All analyses were performed in R software (4.2.2).

The study was done in accordance with standards of reporting quantitative benefit-risk models applied to vaccines (BRIVAC) guideline.^40^

### Ethics statement

The study did not require informed consent or ethical approval, as we used all published data.

### Role of the funding source

The funder of the study had no role in study design, data collection, data analysis, data interpretation, or writing of the report.

## Results

We included 8 RCTs, with 8847 participants, contributing to the input data for the benefit-harm analysis (Table 1 and Appendix Supplementary Fig. S1). The follow-up time in these RCTs ranged from 52 weeks to 104 weeks, with the majority followed up for about one year. The participants were predominately women (74.1%) and people living with obesity (96.0%), with a pooled average age of 46.7 years.

**Table 1:** Trial and aggregated characteristics at baseline.

Study	Follow-up	BMI	GLP-1 receptor agonist	Placebo	Adjunct interventions in both GLP-1 receptor agonist and placebo groups	Sample size	% Women	Age (SD)	BMI (SD)	BMI ≥ 30, %
Astrup et al. 2012^4^	52 weeks	30 to 40	3 mg liraglutide daily	Placebo daily	Caloric deficit: 500 kcal/day Increased physical activity	98 93	75.0 75.0	45.9 (10.3) 45.9 (10.7)	34.9 (2.8) 34.8 (2.8)	100.0 100.0
Pi-Sunyer et al. 2015^36^	56 weeks	≥27 + dyslipidaemia or hypertension ≥30	3 mg liraglutide daily	Placebo daily	Caloric deficit: 500 kcal/day Physical activity: 150 min/week Counselling sessions: approximately every 4 weeks	1244 2487	78.1 78.7	45 (12.0) 45.2 (12.1)	38.3 (6.3) 38.3 (6.4)	96.46 97.35
O’Neil et al. 2018^34^	52 weeks	≥30	3 mg liraglutide daily	Placebo daily	Caloric deficit: 500 kcal/day Physical activity: 150 min/week Counselling sessions: approx. every 4 weeks	136 103	65.0 65.0	46 (13.0) 49 (11.0)	40.1 (7.2) 38.6 (6.6)	100 100
Wilding et al. 2021^41^	68 weeks	≥27 + dyslipidaemia, hypertension, obstructive sleep apnoea, CVD ≥30	2.4 mg semaglutide weekly	Placebo weekly	Caloric deficit: 500 kcal/day Physical activity: 150 min/week Counselling sessions: approx. every 4 weeks	655 1306	76.0 73.1	47 (12.0) 46 (13.0)	38 (6.5) 37.8 (6.7)	94.50 93.80
Garvey et al. 2022^42^	104 weeks	≥27 + dyslipidaemia, hypertension, obstructive sleep apnoea, CVD ≥30	2.4 mg semaglutide weekly	Placebo weekly	Caloric deficit: 500 kcal/day Physical activity: 150 min/week Counselling sessions: approximately every 4 weeks	152 152	74.3 80.9	47.4 (10.3) 47.3 (11.7)	38.5 (7.2) 38.6 (6.7)	
Jastreboff et al. 2022^5^	72 weeks	≥27 + dyslipidaemia, hypertension, obstructive sleep apnoea, CVD ≥30	10 mg and 15 mg tirzepatid weekly	Placebo weekly	Caloric deficit: 500 kcal/day Physical activity: 150 min/week Counselling sessions: approximately every 8 weeks	643 636 630	67.8 67.1 67.5	44.4 (12.5) 44.7 (12.4) 44.9 (12.3)	38.2 (6.89) 38.2 (7.01) 38.2 (6.89)	96.27 94.03 93.65
Rubino et al. 2022^43^	68 weeks	≥27 + dyslipidaemia, hypertension, obstructive sleep apnoea, CVD ≥30	3 mg liraglutide daily 2.4 mg semaglutide weekly	Placebo daily Placebo weekly	Caloric deficit: 500 kcal/day Physical activity: 150 min/week Counselling sessions: approximately every 4–6 weeks	85 126 127	77.6 81.0 76.4	51 (12.0) 49 (13.0) 48 (14.0)	38.8 (6.5) 37.2 (6.4) 37 (7.4)	95.29 91.34 92.86
Wharton et al. 2023^44^	104 weeks	≥30 or ≥27 with ≥1 weight-related comorbidity except diabetes	Semaglutide (2.4 mg) weekly	Placebo daily	500 kcal deficit per day 150 min of physical activity per week	88 86	80.7 74.4	47.3 (12.3) 47.3 (10.7)	39.3 (6.9) 38.1 (7.9)	97.7 97.7

The treatments and placebo were administered subcutaneously.

BMI: Body mass index, SD: Standard deviation, IQR: Interquartile range, CVD: Cardiovascular disease, GLP-1 RA: Glucagon-like peptide-1 receptor agonist.

The summary treatment effects of GLP-1 RAs are shown in Table 2 (see detailed results in Appendix Supplementary Fig. S2). The number of individuals achieving a weight loss target of ≥5% and ≥10% from baseline was significantly higher in the GLP-1 RA group than placebo, with a relative risk (RR) of 2.51 (95% CI 2.37–2.66) and 4.11 (3.45–4.90), respectively. The effects were qualitatively consistent across the specific GLP-1 RA analogues. Semaglutide showed a relatively larger effect (RR 5.42, 4.53–6.50), followed by tirzepatide (4.29, 3.65–5.05) and liraglutide (2.91, 2.19–3.87) in achieving the 10% weight loss target. The magnitude of the effect on weight loss of 5% was also in a similar order.

**Table 2:** Relative effects and baseline rates of GLP-1 receptor agonists.

Outcomes	Combined GLP-1 RAs	Semaglutide	Liraglutide	Tirzepatide	Annual events in 1000 GLP-1 RA-untreated people	Preference weights
	RR (95% CI)	RR (95% CI)	RR (95% CI)	RR (95% CI)	Incidence (95% CI)	Value (range)
Weight loss ≥5%	2.51 (2.37–2.66)	2.63 (2.38–2.91)	2.36 (2.17–2.58)	2.60 (2.34–2.90)	236 (222–249)	0.10 (0.05–0.15)
Weight loss ≥10%	4.11 (3.45–4.90)	5.42 (4.53–6.50)	2.91 (2.19–3.87)	4.29 (3.65–5.05)	98 (88–107)	0.25 (0.20–0.30)
Abdominal pain	1.78 (1.37–2.30)	2.66 (1.01–6.97)	1.62 (1.17–2.24)	1.57 (0.97–2.55)	28 (22–33)	0.05 (0.00–0.10)
Alopecia	5.67 (2.47–13.00)	–	–	5.67 (2.47–13.00)	5 (1–8)	0.25 (0.20–0.30)
Cholecystitis	12.00 (1.62–88.85)	–	4.06 (0.27–61.60)	8.64 (0.50–149.41)	0 (0–0.75)	0.25 (0.20–0.30)
Cholelithiasis	1.90 (1.12–3.22)	2.73 (1.15–6.50)	1.89 (0.76–4.67)	1.10 (0.42–2.88)	4 (2–7)	0.25 (0.20–0.30)
Constipation	2.31 (1.81–2.94)	2.27 (1.72–3.00)	2.10 (1.21–3.67)	2.51 (1.79–3.53)	53 (47–60)	0.05 (0.00–0.10)
Diarrhoea	1.85 (1.37–2.50)	1.54 (1.09–2.17)	1.57 (0.89–2.75)	3.03 (2.26–4.06)	70 (62–77)	0.05 (0.00–0.10)
Dizziness	1.57 (1.10–2.25)	–	1.39 (1.05–1.86)	2.07 (1.18–3.60)	19 (14–24)	0.10 (0.05–0.15)
Dyspepsia	2.77 (2.26–3.40)	2.69 (1.88–3.85)	2.90 (2.14–3.98)	2.50 (1.67–3.74)	26 (21–31)	0.05 (0.00–0.10)
Eructation	5.62 (2.18–14.50)	5.64 (1.04–30.71)	2.19 (0.25–19.42)	8.63 (3.16–23.56)	4 (1–7)	0.05 (0.00–0.10)
Fatigue	1.47 (1.12–1.91)	2.02 (0.68–6.07)	1.47 (1.13–1.90)	–	45 (35–55)	0.10 (0.05–0.15)
Flatulence	2.09 (1.05–4.16)	2.00 (0.97–4.13)	3.16 (0.33–29.85)	–	21 (7–36)	0.05 (0.00–0.10)
Headache	1.12 (0.98–1.27)	1.22 (0.98–1.52)	1.09 (0.92–1.28)	1.02 (0.71–1.45)	61 (54–68)	0.10 (0.05–0.15)
Hypoglycaemia	2.89 (1.28–6.52)	1.82 (0.19–17.12)	4.22 (2.28–7.81)	10.16 (1.37–75.52)	3 (0.3–6)	0.10 (0.05–0.15)
Injection site reactions	1.44 (0.48–3.32)	0.55 (0.20–1.54)	1.53 (0.81–2.90)	16.51 (4.05–67.20)	3 (1–6)	0.10 (0.05–0.15)
Nausea	2.72 (2.48–2.98)	2.55 (2.20–2.95)	2.70 (2.38–3.07)	3.39 (2.63–4.36)	89 (81–98)	0.05 (0.00–0.10)
Pancreatitis	1.67 (0.46–6.05)	3.51 (0.18–67.89)	2.09 (0.35–12.26)	1.02 (0.09–11.18)	0.2 (0–1.0)	0.25 (0.20–0.30)
Upper abdominal pain	1.77 (1.31–2.39)	2.20 (1.08–4.49)	1.80 (0.98–3.31)	–	29 (21–37)	0.05 (0.00–0.10)
Vomiting	4.16 (3.47–4.98)	4.10 (3.13–5.36)	3.84 (2.97–4.97)	6.70 (3.65–12.27)	19 (15–23)	0.05 (0.00–0.10)
Discontinuation due to adverse events	2.17 (1.73–2.71)	1.94 (1.32–2.86)	2.13 (1.35–3.35)	2.51 (1.50–4.19)	27 (22–32)	0.25 (0.20–0.30)

These results were used as input data for the benefit-harm balance analysis. GLP-1 = Glucagon-like Peptide-1. RR: relative risk.

Events <1 per 1000 reported with decimal points to avoid overestimation due to rounding.

The harm events were significantly higher in GLP-1 RA-treated participants, including abdominal pain (RR 1.78, 1.37–2.30), constipation (2.31, 1.81–2.94), diarrhoea (1.85, 1.37–2. 50), eructation (5.62, 2.18–14.50), hypoglycemia (2.89, 1.28–6.52), nausea (2.72, 2.48–2.98), and vomiting (4.16, 3.47–4.98). Moderate harm outcomes such as cholelithiasis (1.90, 1.12–3.22), alopecia (5.67, 2.47–13.00) and cholecystitis (12.00, 1.62–88.85) were also higher in the GLP-1 RA group, while pancreatitis (1.87, 0.52–6.67) was not statistically different between the treatment groups. Although the incidence of most harm outcomes was consistent across the specific GLP-1 RAs, some were specific, such as alopecia (observed in tirzepatide) and cholecystitis (observed only in tirzepatide and liraglutide), leading to between-treatment variations in net benefit (Table 2).

Treatment discontinuation due to adverse effects was also more than twice higher in the GLP-1 RA-treated individuals than the placebo (RR 2.17, 1.73–2.71).

### Benefit-harm balance

#### Combined GLP-1 RAs

**Fig. 1:**
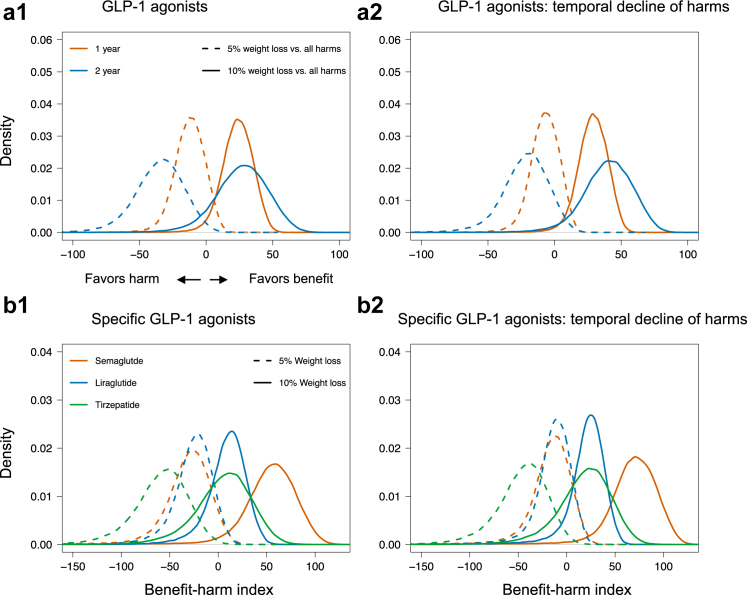
Benefit harm balance of weight loss versus all harm outcomes. (a1) Combined GLP-1 RAs, (a2) Combined GLP-1 RAs, where decreasing rates over time was assumed for some harm outcomes, (b1) Specific GLP-1 treatments over 2 years (b2) Specific GLP-1 RA treatments over 2 years, where decreasing rates over time was assumed for some harm outcomes (see methods). A positive index shows more benefits than harms and a negative index shows more harms than benefits and zero shows that benefit equals harms.

The expected outcome differences and benefit-harm results are presented in Fig. 1 and Table 3. As shown in Fig. 1 a1, achieving a 10% weight loss was net beneficial for the combined GLP-1 RAs over 1 and 2 years of treatment, with positive average indices and corresponding probabilities of 0.97 and 0.91, respectively. The absolute net benefit of achieving 10% weight loss in 2 years without experiencing worrisome harms was 104 (95% CI 100–112) per 1000 people. However, the benefit of achieving a 5% weight loss did not exceed the cumulative harms at both time horizons.

**Table 3:** Outcome probability differences and benefit-harm balance of GLP-1 receptor agonists over 2 years of treatment.

Benefit-harm balance	Combined GLP-1 RAs Events per 1000 people (95% CI)	Semaglutide Events per 1000 people (95% CI)	Liraglutide Events per 1000 people (95% CI)	Tirzepatide Events per 1000 people (95% CI)
Absolute net benefit of achieving 10% weight loss^a^	104 (100, 112); Prob. = 0.91^b^	208 (204, 220); Prob. = 0.96	32 (28, 40); Prob. = 0.72	20 (12, 28); Prob. = 0.60
Absolute net benefit of achieving 5% weight loss^a^	−144 (−148, −140); Prob. = 0.01	−132 (−140, −124); Prob. = 0.06	−104 (−112, −100); Prob. = 0.07	−240 (−244, −232); Prob. = 0.00
**Outcome differences compared to placebo over 2 years**				
≥10% weight loss	375 (352, 399)	476 (438, 515)	257 (228, 287)	391 (348, 434)
≥5% weight loss	318 (296, 339)	335 (298, 370)	295 (263, 327)	330 (291, 369)
Abdominal pain	41 (19, 69)	98 (1, 273)	34 (9, 65)	32 (−2, 80)
Alopecia	57 (9, 176)			57 (9, 176)
Cholecystitis	0 (0, 0)	–	0 (0, 0)	0 (0, 0)
Cholelitiasis	8 (1, 21)	17 (1, 50)	9 (−2, 33)	2 (−5, 16)
Constipation	118 (78, 164)	115 (66, 175)	105 (20, 223)	136 (72, 214)
Diarrhoea	100 (42, 173)	66 (11, 137)	73 (−13, 196)	217 (131, 321)
Dizziness	22 (4, 46)	–	15 (2, 32)	41 (7, 95)
Dyspepsia	84 (58, 117)	82 (41, 135)	91 (52, 140)	74 (32, 129)
Eructation	46 (7, 144)	59 (0, 258)	25 (−8, 160)	74 (12, 228)
Fatigue	39 (10, 75)	99 (−27, 337)	39 (11, 74)	
Flatulence	51 (2, 152)	54 (−14, 214)	150 (−30, 712)	–
Headache	13 (−2, 29)	24 (−2, 55)	10 (−8, 30)	4 (−32, 48)
Hypoglycemia	17 (1, 68)	18 (−9, 134)	26 (3, 96)	101 (2, 507)
Injection site reaction	4 (−3, 19)	−2 (−7, 3)	4 (−1, 14)	115 (16, 374)
Nausea	221 (191, 252)	202 (159, 248)	219 (180, 261)	291 (210, 379)
Pancreatitis	4 (−2, 39)	23 (−3, 246)	8 (−2, 71)	4 (−9, 40)
Upper abdominal pain	43 (15, 87)	72 (4, 190)	48 (−1, 132)	–
Vomiting	110 (80, 145)	109 (71, 155)	100 (66, 142)	195 (90, 344)

GLP-1 = Glucagon-like Peptide-1.

a Incidence of achieving a 10% (or 5%) weight loss per 1000 people over 2 years without worrisome harms.

b Probability that the absolute net benefit exceeds zero or that the benefit outweighed the harms.

In the sensitivity analysis assuming decreasing rates for harm outcomes (see methods), the probability of net benefit generally improved, as reflected in Fig. 1 a2. A 10% weight loss outweighed harms at both 1- and 2- year horizons with probabilities of 0.99 and 0.97, respectively. Yet the benefit of a 5% weight loss did not yet exceed the probability threshold at both the time horizons, regardless of some increase in benefit.

#### Specific GLP-1 RAs

The net benefit was found to differ between specific GLP-1 RAs. We present the results in Fig. 1 b1 focusing on the 2-year time horizon (see results over the 1-year horizon in Appendix Supplementary Table S1). The 10% weight loss was net beneficial for all specific treatments; however, similar to the combined GLP-1 RAs, achieving a 5% weight loss did not exceed the harms for any of the specific treatments. The probabilities of net benefit for the 10% weight loss target were 0.96, 0.72, and 0.60 at 2 years of treatment, respectively for semaglutide, liraglutide, and tirzepatide. The corresponding absolute net benefit were estimated to be 208 (204–220), 32 (28–40), and 20 (12–28) per 1000 people achieving the 10% weight loss without experiencing any worrisome harms.

In the alternative analysis in which we assumed decreasing rates for some of the harm outcomes over time, the net benefit generally showed an improvement, albeit minimal compared to results with constant rates (Fig. 1 b2).

### Preference-sensitivity

Weight loss was the most influential outcome on the net benefit of GLP-1 RAs, followed by side effects like nausea, diarrhoea, constipation, and vomiting, which were relatively frequent. Most moderately severe harm outcomes had less impact on the net benefit due to their lower frequency and/or less precise effects (Appendix Supplementary Fig. S3).

In assessing preference sensitivity, we varied preferences (from 0 to 0.5) that most people may have rather than using an average value. We performed this analysis for selected outcomes, specifically benefit outcome (weight loss), more frequent harms (nausea and headache), and cholelithiasis (moderately severe harm outcome), while keeping the weights of the others unchanged.

**Fig. 2:**
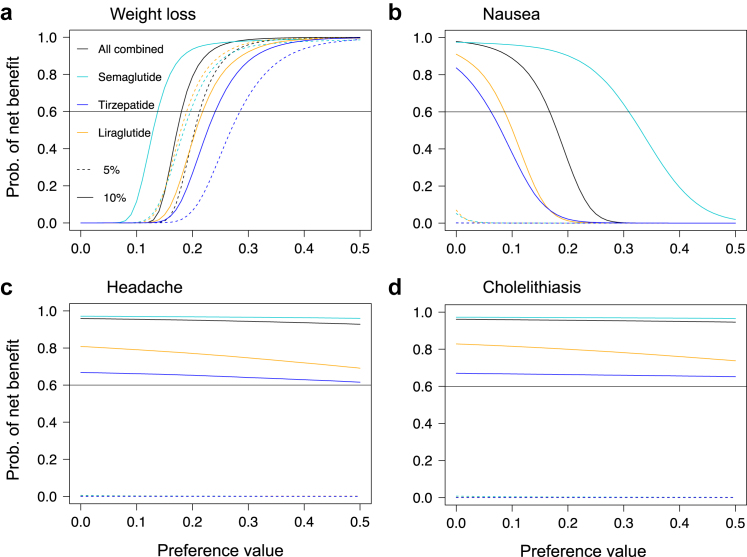
Preference-sensitivity of benefit-harm assessment. Net benefit according to range of preferences over 2-years of treatments for weight loss and selected harm outcomes.

Fig. 2 illustrates that individuals derive a net benefit from GLP-1 RAs, like semaglutide, liraglutide, and tirzepatide, even for the 5% weight loss target, when the preference for weight reduction increased. For instance, semaglutide, liraglutide, and tirzepatide met the 0.6 probability threshold for net benefit at preference weights of 0.20, 0.19, and 0.28 for achieving a 5% weight loss and at preference weights of 0.14, 0.22, and 0.24 for achieving a 10% weight loss (Fig. 2a). However, the net benefit decreased significantly when greater weight (i.e., concern) was placed on harm outcomes such as nausea due to their high frequency (Fig. 2b). In contrast, headaches, despite being frequent, had a smaller relative effect, thus less impact on net benefit (Fig. 2c). Similarly, cholelithiasis, while moderately severe and relatively impactful, was a rare outcome and thus did not significantly affect the net benefit (Fig. 2d). Although not shown in the plots, cholecystitis also had no impact on the benefit harm balance for similar reasons.

### Benefit-harm balance of weight loss versus discontinuation due to harm outcomes

When accounting for all-cause discontinuation (instead of specific harm outcomes), the benefit-harm balance analysis showed that the probabilities of achieving a 10% weight loss with GLP-1 RAs, including semaglutide, liraglutide, and tirzepatide, were all 1.00. For a 5% weight loss goal, the respective probabilities were 1.00 for semaglutide, 1.00 for liraglutide, 0.98 for tirzepatide, and 0.65 when considering all GLP-1 RAs combined.

## Discussion

Our analyses show that GLP-1 RAs used for weight reduction in people with overweight and obesity result in a net benefit in achieving a 10% weight loss during the initial phases of treatment, particularly in the first and second year. The 5% weight loss was not large enough to outweigh the harms associated with the treatments; however, it could be net beneficial for some individuals, depending on their willingness to accept the harm outcomes.

Most GLP-1 RA harms were mild and could decrease over the duration of treatment. Assuming a contant rate may thus result in an underestimation of the net benefit. This was partially exhibited in some of the included RCTs, wherethe most common adverse effects occurred during the initiation of the treatment and decreased over time.^34^, ^35^, ^36^, ^37^, ^38^, ^39^ We accounted for this declining rates of harms in our benefit-harm analysis, mainly for those associated with tolerance and adaptation problems, as well as the placebo effect.^45^ However, despite this adjustment, the net benefit of achieving a 5% weight loss did not show any important improvement. It is important to note that these findings are specific to individuals strictly adhering to lifestyle changes, including exercise and diet.

We also explored differences between specific GLP-1 RAs. Tirzepatide exhibited less favourable overall benefit compared to semaglutide and liraglutide as some harm outcomes were observed more frequently in the tirzepatide therapy, such as hypoglycemia, injection site reactions, and alopecia. It is important to note that the analysis of tirzepatide for patients living without diabetes was restricted to one study, namely the SURMOUNT-1 trial. The findings may not be conclusive.^5^

Another important finding of our study was the sensitivity of the net benefit to preferences. The net benefit was found to be highly dependent on patient preferences, i.e., the individual’s willingness to accept harms (or their risk aversion) to achieve weight reduction. Even the 5% weight loss could be net beneficial for individuals who place greater preference on the weight reduction and less concern on the potential harms. For example, one of the harm outcomes that highly influenced the unfavourable benefit-harm balance of tirzepatide was alopecia. This may not be a concern at all for individuals who no longer have hair. They could choose tirzepatide over other GLP-1 RAs, due to its more pronounced effect on weight loss. The between-individual variation of net benefit could also be much higher than that shown in this study if we simultaneously consider individual preferences for all outcomes. The preference-sensitivity of the net benefit underscores the importance of personalised treatment decisions considering individual patients' preferences and values, in addition to their outcome risks and treatment goals. This would help to optimise the benefits, reduce treatment (mis)overuse, and improve resource allocation for those who truly need the treatments. Such decisions could be enhanced by extending the benefit-harm approach to develop decision support aids. Although it is beyond the scope of this study, a companion project within the LOOBesity cohort at the University of Zurich and University Hospital of Zurich is developing a benefit–benefit decision aid aimed at personalized treatments for individual patients.^46^

The trade-off between the benefits and harms of GLP-1 RA treatment is challenging due to the multitude of parameters and outcomes to consider concurrently. But it is even more challenging when considering GLP-1 RAs for weight maintenance. The current evidence suggests that GLP-1 RAs may serve as an effective treatment strategy for rapid weight loss in conjunction with lifestyle interventions during the first years of treatment. However, all RCTs showed that weight reduction stablizes over time after the initial 1 or 2 years of treatment. This triggers an important question: whether discontinuing (but having a high risk of weight rebounding) or continuing GLP-1 RA therapy for weight maintenance (with no further weight loss but higher harm risks) is beneficial for the overall health of individuals, which needs a separate analysis. For instance, the STEP 1 and STEP 4 trials demonstrated that patients who stopped taking semaglutide experienced significant weight regain and a reversal of cardiometabolic benefits, whereas those who continued the treatment maintained their weight loss.^47^^,^^48^ Similarly, the S-LITE and SURMOUNT-4 trials also reported weight regain following the discontinuation of liraglutide and tirzepatide, respectively.^49^^,^^50^ This was supported by an observational study, albeit in people living with diabetes, that reported an increased risk of CVD events in those who withdrew GLP-1 RAs.^51^

The long-term harms and benefits are also not clear. Although they are too short to observe long-term benefits or harm outcomes, the RCTs available so far have signaled some important harms, including pancreatitis, renal failure, and alopecia, which warrant close monitoring. Long-term data, such as the LOOBesity cohort,^46^ and post-marketing surveillance data, are needed to better understand the long-term benefits and harms. On the other hand, GLP-1 RA therapy may also have a long-term positive effect on preventing weight-related comorbidities such as CVD. The SELECT trial, revealed that semaglutide reduced the risk of recurrent CVD events by 20%.^13^ This supports the findings in the diabetic population showing the preventive benefit of GLP-1 RAs for CVD and related conditions.^12^^,^^52^^,^^53^ This points to a promising future for GLP-1 RAs in CVD prevention, potentially tipping the benefit-harm trade-off in favour of a net benefit.

These uncertainties remind us the need for a critical and continuous update of the benefit-harm balance with the rapidly evolving evidence. The current study is dedicated to proactively updating, synthesizing, and monitoring the benefit-harm balance over time. This includes updating the evidence on the essential parameters needed for the analysis, such as effectiveness and safety, baseline outcome probabilities from more broader target populations, and outcome preferences. The continous analysis provides decision-makers up-to-date guidance on the potential of GLP-1 RAs for acute and long-term weight management and CVD prevention, as well as early the detection of unncessary harms.

We performed the first comprehensive assessment of the benefit-harm balance, particularly for the initiation of GLP-RAs for rapid weight loss reduction. However, it is important to acknowledge some of the limitations of the study. First, because the drugs analysed are relatively new indications in the treatment of overweight and obesity, we could not find sufficient evidence for all the harm outcomes chosen in the analysis. Another limitation was that the selection of harm outcomes might have involved some subjectivity. Too many adverse effects were reported across the RCTs, but we narrowed our selection according to the Swiss drug compendium to focus on the most frequent (>1%) or more serious ones regardless of their frequency.^21^, ^22^, ^23^ However, outcomes that were excluded because of inconsistent reporting or infrequent incidence in the source RCTs might, in fact, be important harms and manifest in long-term observations. In addition, our modelling study used control arms of RCTs for the source of outcome probability distributions in untreated individuals. This was because we were unable to find data representative of people eligible for GLP-1 RA treatments in the general population. Thus, the outcome risks from the RCTs may not be well reflective of the true risk distributions in the general population with overweight or obesity. Lastly, the absence of empirical preferences might be considered a limitation. Nonetheless, our analysis probed its influence, which demonstrated that aggregate preferences are not applicable due to the high sensitivity of the benefit-harm balance to personal preference variations.

This first quantitative benefit-harm balance analysis showed that GLP-1 RAs derive a net benefit for achieving a weight reduction of at least 10% from baseline over the first and second years of treatment. The benefit did not exceed the harms for a 5% weight loss target; however, it could be net beneficial for some individuals, depending on their willingness to accept harms in pursuit of weight loss, implying the need for patient-tailored decisions to maximise the benefits and minimize harms and overuse of treatments. As additional evidence on the benefit and harm effects of GLP-1 RAs, patient preferences, and outcome risks emerge, the benefit-harm balance of GLP-1 RAs should be regulary updated.

## Contributors

HM and EF are joint corresponding authors and contributed equally to this work. MAP and HGY conceived and designed the study. HM, EF, and HY acquired and analysed the data and prepared the manuscript. HM, EF, HGY, MAP, PG, BG, MK, JB, and FB critically revised the manuscript for important intellectual content and approved the final manuscript. HM, EF, and HGY accessed and verified the data. HGY, HM, and EF attested that all listed authors meet authorship criteria and that no others meeting the criteria have been omitted. HGY and MAP are the guarantors of this manuscript.

## Data sharing statement

All data are published and available in the article.

## Declaration of interests

PG received payment as a speaker at symposia from Novo Nordisk and Eli Lilly. Other authors declare no competing interests.
