# Corrigendum to “GLP-1 receptor agonists for weight reduction in people living with obesity but without diabetes: a living benefit-harm modelling study” eClinicalMedicine 2024;73:102661

**DOI:** 10.1016/j.eclinm.2026.104022

**Published:** 2026-06-24

**Authors:** Hannah Moll, Eliane Frey, Philipp Gerber, Bettina Geidl, Marco Kaufmann, Julia Braun, Felix Beuschlein, Milo A. Puhan, Henock G. Yebyo

**Affiliations:** a Department of Epidemiology, Epidemiology, Biostatistics and Prevention Institute, University of Zurich, Zurich, Switzerland; b Department of Chemistry and Applied Biosciences, Institute of Pharmaceutical Sciences, ETH Zurich, Zurich, Switzerland; c Department of Endocrinology, Diabetology and Clinical Nutrition, University Hospital Zurich and University of Zurich, Zurich, Switzerland; d Medizinische Klinik und Poliklinik IV, Klinikum der Universität, Ludwig-Maximilians-Universität, Munich, Germany; e The LOOP Zurich - Medical Research Center, Zurich, Switzerland

## Description of the error and impact on results and conclusions

After publication, we identified that Wharton et al. (2023) had been erroneously included among the randomised controlled trials (RCTs) informing the semaglutide evidence base (doi.org/10.1002/oby.23673). We sincerely regret this error and have corrected the analyses accordingly. The publication was a secondary analysis of the STEP 5 trial, whose primary report by Garvey et al. (2022) was included in the analysis. This inclusion therefore unintentionally double-counted the same trial population. Wharton et al. (2023) has been removed, and the affected analyses have been rerun using the remaining 7 RCTs. The changes to the estimates were small and did not alter the findings, conclusions, or overall interpretation.

All figures, tables, and supplementary materials were reviewed. Those directly affected by the removed publication were corrected as shown below. The corrections were cross verified by Hannah Moll and Henock G. Yebyo.

## Corrections to the summary

While the overall findings and interpretation remain unchanged, the results affected by the removal of Wharton et al. (2023) should read as follows:

“*We included 7 RCTs involving 8673 participants. The pooled average age was 45.6 years, with the majority being women (74.6%) and people living with obesity (95.8%).*”

“*Achieving a 10% weight loss with GLP-1 RA therapy outweighed the cumulative harms, with a net benefit probability of 0.96 at year 1 and 0.88 at year 2. The absolute net benefit was equivalent to 100 (92 to 104) per 1000 persons achieving a 10% weight loss over 2 years without experiencing any worrisome harm*. *A 5% weight loss did not show a net benefit, with probabilities of 0.14 and 0.01at year 1 and year 2, respectively*.”

## Corrections to the research in context panel

No changes.

## Corrections to the main text

Following the removal of Wharton et al. (2023), the number of RCTs was reduced from 8 to 7, with a corresponding reduction in the total number of participants. The meta-analytic point estimates changed only minimally. For 5% and 10% weight loss, respectively, the estimates changed from 2.63 to 2.65 and from 5.42 to 5.41 for semaglutide, and from 2.51 to 2.52 and from 4.11 to 4.03 for all GLP-1 RAs (see revised Table 1 below). The revised text in the results section of the original article should read as follows:

“*We included 7 RCTs, with 8673 participants, contributing to the input data for the benefit-harm analysis. The participants were predominately women (74.6%) and people living with obesity (95.8%), with a pooled average age of 45.6 years*. *The summary treatment effects of GLP-1 RAs are shown in Table 2 (see detailed results in Appendix Supplementary Fig. S2). The number of individuals achieving a weight loss target of ≥5% and ≥10% from baseline was significantly higher in the GLP-1 RA group than placebo, with a relative risk (RR) of 2.52 (95% CI, 2.38–2.67) and 4.03 (3.35–4.85), respectively. The effects were qualitatively consistent across the specific GLP-1 RA analogues. Semaglutide showed a relatively larger effect (RR 5.41, 4.48–6.54), followed by tirzepatide (4.29, 3.65–5.05) and liraglutide (2.91, 2.19–3.87) in achieving the 10% weight loss target.*”

These revisions affect only the overall GLP-1 RAs and semaglutide estimates. Estimate for the other GLP-1 RA types remain unchanged.

## Corrections to figures

The shapes of the benefit–harm balance distributions remain nearly identical to those in the original analysis.

**Figure:**
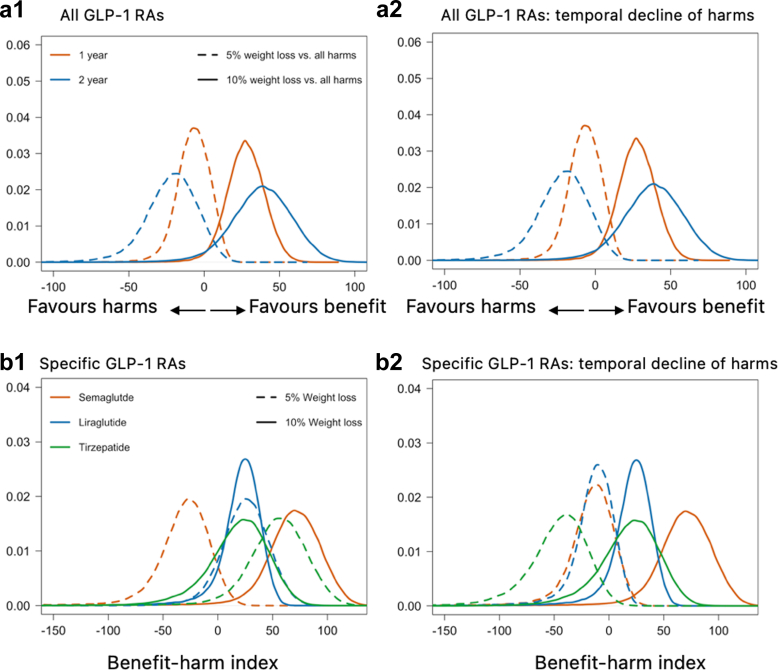

## Correction to tables

**Table 1: Trial and aggregated characteristics at baseline**.

In Table 1, Wharton et al., 2023 should be disregarded; all other entries remain unchanged.

**Table 2: Relative effects and baseline rates of GLP-1 receptor agonists**.

The revised estimates affected by the removal of Wharton et al. (2023) are shown below; all other estimates in the original table remain unchanged.

**Table:** 

Model	Original	Corrected
Effect of semaglutide on 5% weight loss	2.63 (2.38–2.91)	2.65 (2.34–3.00)
Effect of semaglutide on 10% weight loss	5.42 (4.53–6.50)	5.41 (4.48–6.54)
All GLP-1 RAs on 5%	2.51 (2.37–2.66)	2.52 (2.38–2.67)
All GLP-1 RAs on 10%	4.11 (3.45–4.90)	4.03 (3.35–4.85)

## Benefit-harm balance

### Combined GLP-1 RAs

Following replacement of GLP-1 RA effects with the corrected estimates, the 2-year probability of net benefit changed from 0.91 to 0.88 in the main analysis, as shown in Table 2, and from 0.97 to 0.96 in the sensitivity analysis. The Results section with the correct estimates should thus read as follows:

“*As shown in Fig. 1 a1, achieving a 10% weight loss was net beneficial for the combined GLP-1 RAs over 1 and 2 years of treatment, with positive average indices and corresponding probabilities of 0.96 and 0.88, respectively. The absolute net benefit of achieving 10% weight loss in 2 years without experiencing worrisome harms was 100 (95% CI 92–104) per 1000 people. In the sensitivity analysis assuming decreasing rates for harm outcomes (see methods), the probability of net benefit generally improved, as reflected in Fig. 1 a2. A 10% weight loss outweighed harms at both 1- and 2- year horizons with probabilities of 0.98 and 0.96, respectively*.”

### Specific GLP-1 RAs

The 2-year probability of net benefit for semaglutide remained unchanged at 0.96, but the absolute net benefit changed from 208 (204–220) to 212 (204–220). The affected section, with the corrected estimates, should read as follows:

“*The probabilities of net benefit for the 10% weight loss target were 0.96, 0.72, and 0.60 at 2 years of treatment, respectively for semaglutide, liraglutide, and tirzepatide. The corresponding absolute net benefits were estimated to be 212 (204, 220), 32 (28–40), and 20 (12–28) per 1000 people achieving the 10% weight loss without experiencing any worrisome harms.”*

**Table 3. Outcome probability differences and benefit-harm balance of GLP-1 receptor agonists over 2 years of treatment**.

**Table:** 

Benefit-harm balance	Combined GLP-1 RAs Events per 1000 people (95% CI)		Semaglutide	
	Original	Corrected	Original	Corrected
Absolute net benefit of achieving 10% weight loss	104 (100, 112); Prob. = 0.91	100 (92, 104); Prob. = 0.88	208 (204, 220); Prob. = 0.96	212 (204, 220); Prob. = 0.96
Absolute net benefit of achieving 5% weight loss	−144 (−148, −140); Prob. = 0.01	−144 (−148, −136); Prob. = 0.01	−132 (−140, −124); Prob. = 0.06	−132 (−140, −124); Prob. = 0.06
Outcome differences compared to placebo over 2 years				
≥10% weight loss	375 (352, 399)	368 (303, 436)	476 (438, 515)	476 (409, 543)
≥5% weight loss	318 (296, 339)	319 (298, 340)	335 (298, 370)	337 (292, 381)

## Corrections to the appendix

The Supplementary Material includes corrections to the figures and tables in the appendix.

