# Supplementary material for: GLP-1 receptor agonists for weight reduction in people living with obesity but without diabetes: a living benefit–harm modelling study
Source: eClinicalMedicine. 2024 May 27;73:102661. doi: 10.1016/j.eclinm.2024.102661 (PMC11154119; doi:10.1016/j.eclinm.2024.102661)
Supplement: Appendix, Supplementary Figs. S1–S3, and Table S1 [file mmc1.pdf]

Caption for Supplementary Material

Appendix 1: Benefit-harm balance model

To assess the 1- and 2-year benefit of GLP-1 RAs, we weighed the cumulative benefits (in terms of achieving a 5% and 10% weight loss, separately) against the cumulative harm outcomes over the same period.[1–6] We predicted the outcome probabilities of achieving the target weight loss and harm outcomes using exponential model as follows:

1. Cumulative probability of achieving weight loss targets or experiencing harm outcomes without taking GLP-1 RA (A=0) was estimated as $p_{i|A=0} = 1000 \times (1 - e^{-I_i \times \mathrm{T}})$; where I_i is baseline risk of each outcome and T is the time horizon.
2. Cumulative probability of achieving weight loss targets or experiencing harm outcomes in people treated with GLP-1 RAs (A=1) was estimated, as $p_{i|A=1} = 1000 \times (1 - e^{-(I_i \times rr_i) \times \mathrm{T}})$; where rr_i is relative effect of GLP-1 agonists vs. placebo.
3. Outcome probability differences of the benefit and harm outcomes were calculated from the above two estimations, as $(p_{i|A=0} - p_{i|A=1})$.
4. To summarize and standardize the benefits and risks in a single number, it is necessary to weigh the outcomes by their respective preference values, as $(p_{i|A=0} - p_{i|A=1}) \times w_i$. where w was the preference weight towards the i outcomes. The preference is a measure of relative importance of the outcomes, which indicates patient's willingness to accept or risk-averse behaviors.
5. The outcome probability differences adjusted by preference weights were aggregated to yield a single benefit-harm balance index (i.e., net clinical benefit), as $\sum_{i=1}^{n}(p_{A=0} - p_{A=1}) \times w$; where w is preference weight and n is number of benefit and harm outcomes related to GLP-1 RA use. The index shows whether achieving weight loss over the time horizons (1 and 2 years) outweighed the harms (positive index) or vice versa (negative index) or shows an equipoise (index equals zero).

The analysis was done stochastically with 100,000 repetitions accounting for the statistical uncertainty of the input estimates to generate a distribution of the net clinical benefit. Interpreting the index can be challenging since it aggregates various outcomes. We provided a proxy interpretation by transforming the index to achieving 10% weight loss. Since we multiplied the outcome probability differences by the respective preference weights (in step 5 above), we converted the index to number of people achieving weight loss, dividing the index by the preference value of 10% weight loss, as $\frac{\sum_{i=1}^{n}(p_{A=0} - p_{A=1}) \times w}{w_{weight\ loss}}$. This can be interpreted as the number of people achieving 10% weight loss without experiencing any worrisome harm outcomes over the selected time horizon.

For a more intuitive interpretation, we emphasized presenting the results in terms of the probability of net benefit. We calculated the probability that the use of GLP-1 RA would have more benefit than harm compared with placebo from the benefit-harm index distribution. The treatment with GLP-1 RA was net beneficial when the probability reached at least 0.60. The point of equipoise (a 0.50 probability of net benefit) is a naïve threshold at which the average expected net benefit equals zero. But we chose 0.6 probability to define the threshold to ensure a non-zero minimal net benefit, since it would not be sensible for patients to initiate a treatment without any benefit, as happens when we take 0.5, where the net benefit is zero.

The analyses included a bootstrapping with 1000 replicate samples to estimate 95% uncertainty intervals based on the 2.5^{th} and 97.5^{th} centiles in the distributions of net benefit and outcome events.

Supplementary Figure 1. Selection of studies for pooling treatment effect of GLP-1 agonists

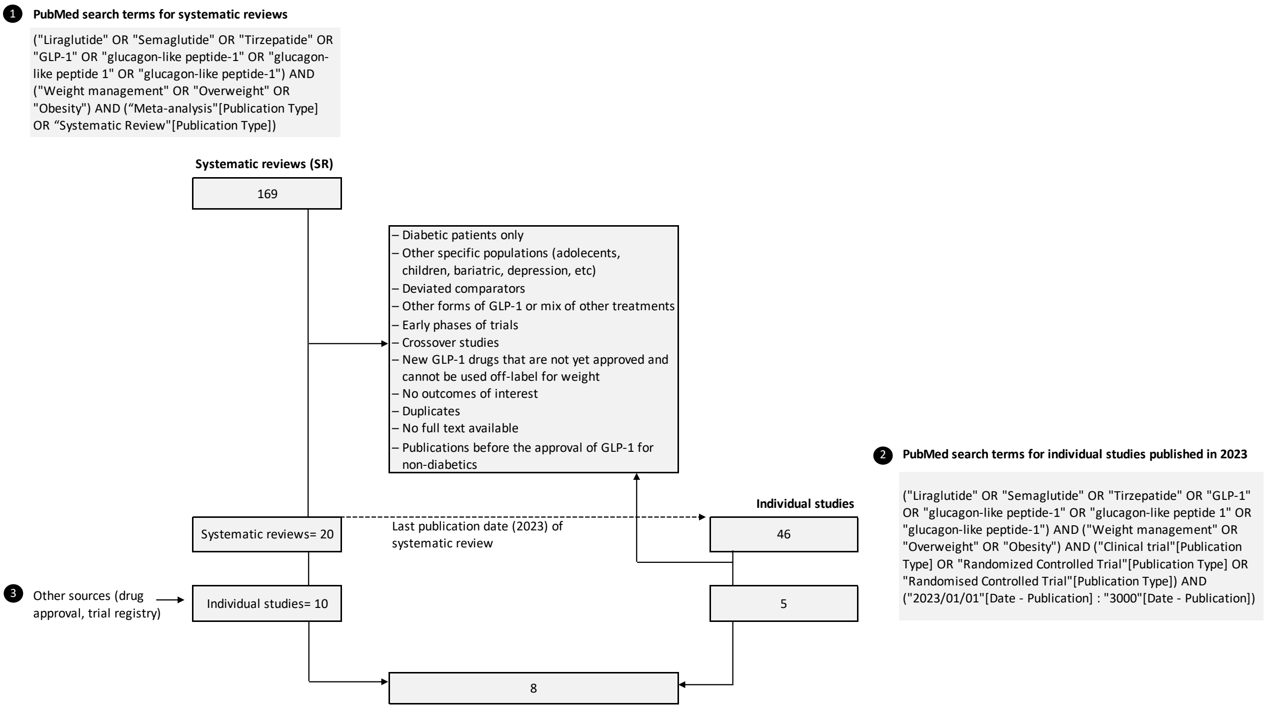

Supplementary Figure 2. Meta-analysis results

5% Weight loss, all GLP-1 agonists

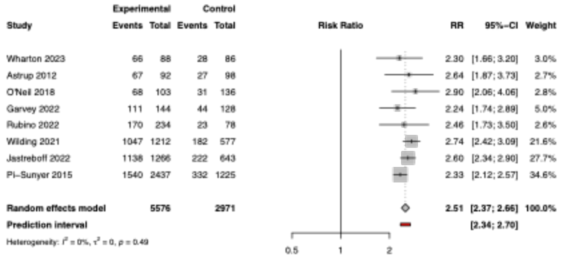

10% Weight loss, all GLP-1 agonists

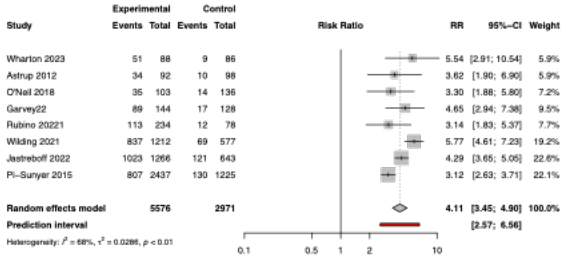

5% Weight loss, semaglutide

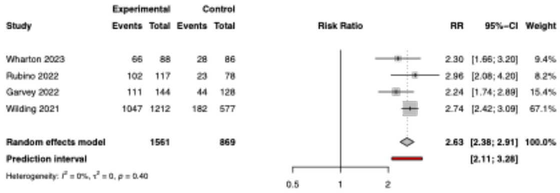

10% Weight loss, semaglutide

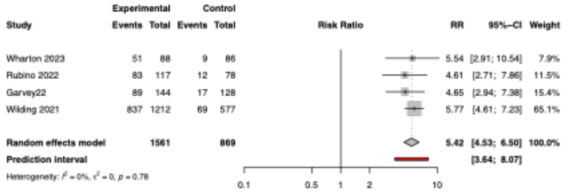

5% Weight loss, liraglutide

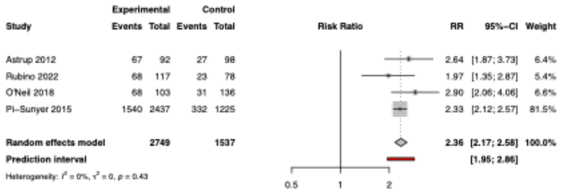

10% Weight loss, liraglutide

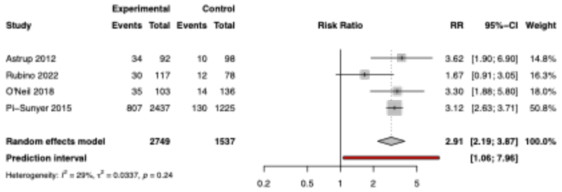

5% Weight loss, tirzepatide

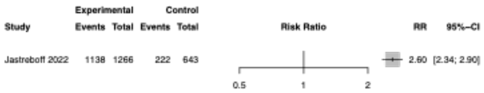

10% Weight loss, tirzepatide

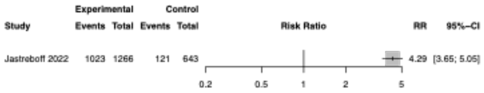

Abdominal pain

Study	Experimental Events	Experimental Total	Control Events	Control Total	RR	95%–CI	Weight
Astrup12	5	93	4	98	1.32	[0.36; 4.76]	3.9%
Garvey22	20	152	4	152	5.00	[1.75; 14.28]	5.8%
Jastreboff22	65	1266	21	643	1.57	[0.97; 2.55]	21.8%
Wilding21	130	1306	36	655	1.81	[1.27; 2.59]	32.9%
Pi–Sunyer15	141	2481	43	1242	1.64	[1.17; 2.29]	35.6%
**Random effects model**		**5298**		**2790**	**1.78**	**[1.37; 2.30]**	**100.0%**
**Prediction interval**						**[0.95; 3.31]**	

Heterogeneity: $I^2 = 9\%$, $\tau^2 = 0.0205$, $p = 0.36$

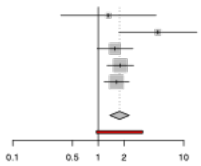

Alopecia

Study	Experimental Events	Experimental Total	Control Events	Control Total	RR	95%–CI
Jastreboff22	67	1266	6	643	5.67	[2.47; 13.00]

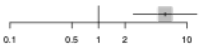

Cholecystitis

Study	Experimental Events	Experimental Total	Control Events	Control Total	RR	95%–CI	Weight
Jastreboff22	8	1266	0	643	8.64	[0.50; 149.41]	49.3%
Pi–Sunyer15	16	2481	0	1242	16.52	[0.99; 275.18]	50.7%
**Random effects model**		**3747**		**1885**	**12.00**	**[1.62; 88.85]**	**100.0%**

Heterogeneity: $I^2 = 0\%$, $\tau^2 = 0$, $p = 0.75$

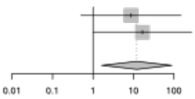

Cholelithiasis

Study	Experimental Events	Experimental Total	Control Events	Control Total	RR	95%–CI	Weight
Garvey22	3	152	2	152	1.50	[0.25; 8.85]	8.9%
Rubino22	3	253	1	85	1.01	[0.11; 9.56]	5.5%
Jastreboff22	13	1266	6	643	1.10	[0.42; 2.88]	30.2%
Wilding21	33	1306	4	655	4.14	[1.47; 11.63]	26.2%
Pi–Sunyer15	20	2481	5	1242	2.00	[0.75; 5.32]	29.3%
**Random effects model**		**5458**		**2777**	**1.90**	**[1.12; 3.22]**	**100.0%**
**Prediction interval**						**[0.80; 4.48]**	

Heterogeneity: $I^2 = 0\%$, $\tau^2 = 0$, $p = 0.43$

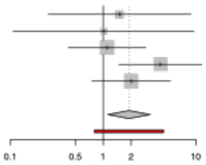

Constipation

Study	Experimental Events	Experimental Total	Control Events	Control Total	RR	95%-CI	Weight
Astrup12	17	93	12	98	1.49	[0.75; 2.95]	8.4%
O'Neil18	24	103	6	136	5.28	[2.24; 12.45]	6.1%
Garvey22	47	152	17	152	2.76	[1.67; 4.59]	12.1%
Rubino22	89	253	20	85	1.50	[0.98; 2.27]	14.5%
Jastreboff22	183	1266	37	643	2.51	[1.79; 3.53]	17.0%
Wilding21	306	1306	62	655	2.48	[1.92; 3.20]	19.9%
Pi–Sunyer15	495	2481	108	1242	2.29	[1.88; 2.79]	22.0%
**Random effects model**		**5654**		**3011**	**2.31**	**[1.81; 2.94]**	**100.0%**
**Prediction interval**						**[1.15; 4.64]**	

Heterogeneity: $I^2 = 42\%$, $\tau^2 = 0.0590$, $p = 0.11$

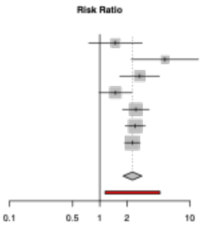

Diarrhoea

Study	Experimental Events	Experimental Total	Control Events	Control Total	RR	95%-CI	Weight
Astrup12	14	93	10	98	1.48	[0.69; 3.16]	8.6%
O'Neil18	29	103	16	136	2.39	[1.38; 4.17]	11.5%
Garvey22	53	152	36	152	1.47	[1.03; 2.11]	14.9%
Rubino22	58	253	22	85	0.89	[0.58; 1.35]	13.7%
Jastreboff22	280	1266	47	643	3.03	[2.26; 4.06]	16.0%
Wilding21	412	1306	104	655	1.99	[1.64; 2.41]	17.6%
Pi–Sunyer15	518	2481	115	1242	2.25	[1.86; 2.73]	17.6%
**Random effects model**		**5654**		**3011**	**1.85**	**[1.37; 2.50]**	**100.0%**
**Prediction interval**						**[0.69; 4.97]**	

Heterogeneity: $I^2 = 78\%$, $\tau^2 = 0.1240$, $p < 0.01$

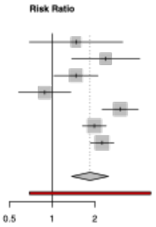

Discontinuation due to harms

Study	Experimental Events	Experimental Total	Control Events	Control Total	RR	95%-CI	Weight
Wharton 2023	8	67	4	69	2.06	[0.65; 6.52]	3.7%
Astrup12	7	93	3	98	2.46	[0.66; 9.23]	2.8%
O'Neil18	9	103	4	136	2.97	[0.94; 9.38]	3.7%
Garvey22	9	152	7	152	1.29	[0.49; 3.36]	5.2%
Rubino22	20	253	3	85	2.24	[0.68; 7.35]	3.5%
Wadden 2022	18	212	18	210	0.99	[0.53; 1.85]	11.5%
Jastreboff22	84	1266	17	643	2.51	[1.50; 4.19]	16.1%
Wilding21	92	1306	20	655	2.31	[1.44; 3.71]	18.3%
Pi–Sunyer15	246	2487	47	1244	2.62	[1.93; 3.55]	35.2%
**Random effects model**		**5939**		**3292**	**2.17**	**[1.73; 2.71]**	**100.0%**
**Prediction interval**						**[1.47; 3.18]**	

Heterogeneity: $I^2 = 14\%$, $\tau^2 = 0.0133$, $p = 0.32$

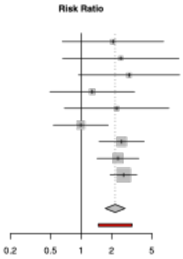

Dizziness

Study	Experimental Events	Experimental Total	Control Events	Control Total	RR	95%-CI	Weight
Jastreboff22	61	1266	15	643	2.07	[1.18; 3.60]	30.9%
Pi–Sunyer15	167	2481	60	1242	1.39	[1.05; 1.86]	69.1%
**Random effects model**		**3747**		**1885**	**1.57**	**[1.10; 2.25]**	**100.0%**

Heterogeneity: $I^2 = 34\%$, $\tau^2 = 0.0264$, $p = 0.22$

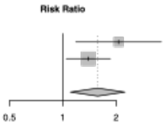

Pancreatitis

Study	Experimental Events	Experimental Total	Control Events	Control Total	RR	95%–CI	Weight
O'Neil18	46	103	24	136	2.53	[1.66; 3.86]	4.8%
Garvey22	81	152	33	152	2.45	[1.75; 3.44]	7.5%
Rubino22	152	253	19	85	2.69	[1.79; 4.04]	5.1%
Jastreboff22	407	1266	61	643	3.39	[2.63; 4.36]	13.4%
Wilding21	577	1306	114	655	2.54	[2.13; 3.03]	27.0%
Pi–Sunyer15	997	2481	183	1242	2.73	[2.37; 3.14]	42.2%
**Random effects model**		**5561**		**2913**	**2.72**	**[2.48; 2.98]**	**100.0%**
**Prediction interval**						**[2.39; 3.10]**	

Heterogeneity: $I^2 = 0\%$, $\tau^2 = 0$, $p = 0.55$

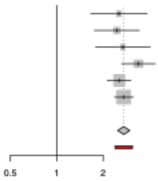

Upper abdominal pain

Study	Experimental Events	Experimental Total	Control Events	Control Total	RR	95%–CI	Weight
Astrup12	5	93	1	98	5.27	[0.63; 44.26]	2.0%
Garvey22	22	152	10	152	2.20	[1.08; 4.49]	17.7%
Pi–Sunyer15	141	2481	43	1242	1.64	[1.17; 2.29]	80.3%
**Random effects model**		**2726**		**1492**	**1.77**	**[1.31; 2.39]**	**100.0%**
**Prediction interval**						**[0.25; 12.36]**	

Heterogeneity: $I^2 = 0\%$, $\tau^2 = 0$, $p = 0.46$

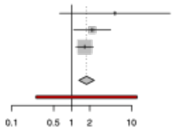

Vomiting

Study	Experimental Events	Experimental Total	Control Events	Control Total	RR	95%–CI	Weight
Astrup12	12	93	2	98	6.32	[1.45; 27.49]	1.5%
O'Neil18	11	103	6	136	2.42	[0.93; 6.33]	3.5%
Garvey22	46	152	7	152	6.57	[3.06; 14.09]	5.6%
Rubino22	58	253	5	85	3.90	[1.62; 9.39]	4.2%
Jastreboff22	145	1266	11	643	6.70	[3.65; 12.27]	8.9%
Wilding21	324	1306	43	655	3.78	[2.79; 5.12]	35.4%
Pi–Sunyer15	404	2481	51	1242	3.97	[2.99; 5.26]	40.8%
**Random effects model**		**5654**		**3011**	**4.16**	**[3.47; 4.98]**	**100.0%**
**Prediction interval**						**[3.28; 5.27]**	

Heterogeneity: $I^2 = 0\%$, $\tau^2 = 0$, $p = 0.45$

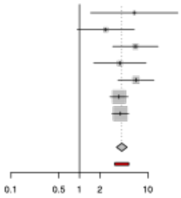

Headache

Study	Experimental Events	Experimental Total	Control Events	Control Total	RR	95%-CI	Weight
O'Neil18	15	103	15	136	1.32	[0.68; 2.58]	3.6%
Garvey22	16	152	16	152	1.00	[0.52; 1.93]	3.7%
Rubino22	38	253	10	85	1.28	[0.67; 2.45]	3.7%
Jastreboff22	84	1266	42	643	1.02	[0.71; 1.45]	12.4%
Wilding21	198	1306	80	655	1.24	[0.97; 1.58]	27.1%
Pi–Sunyer15	327	2481	154	1242	1.06	[0.89; 1.27]	49.5%
**Random effects model**		**5561**		**2913**	**1.12**	**[0.98; 1.27]**	**100.0%**
**Prediction interval**						**[0.93; 1.33]**	

Heterogeneity: $I^2 = 0\%$, $\tau^2 = 0$, $p = 0.88$

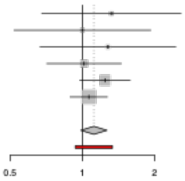

Hypoglycemia

Study	Experimental Events	Experimental Total	Control Events	Control Total	RR	95%-CI	Weight
O'Neil18	2	103	1	136	2.64	[0.24; 28.73]	9.5%
Garvey22	4	152	0	152	9.00	[0.49; 165.73]	6.8%
Rubino22	1	253	0	85	1.01	[0.04; 24.61]	5.8%
Jastreboff22	20	1266	1	643	10.16	[1.37; 75.52]	12.5%
Wilding21	8	1306	5	655	0.80	[0.26; 2.44]	26.4%
Pi–Sunyer15	90	2481	10	1242	4.51	[2.35; 8.63]	39.1%
**Random effects model**		**5561**		**2913**	**2.89**	**[1.28; 6.52]**	**100.0%**
**Prediction interval**						**[0.41; 20.62]**	

Heterogeneity: $I^2 = 46\%$, $\tau^2 = 0.3288$, $p = 0.10$

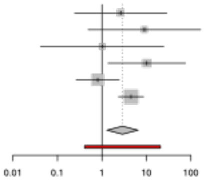

Injection site reactions

Study	Experimental Events	Experimental Total	Control Events	Control Total	RR	95%-CI	Weight
O'Neil18	10	103	10	136	1.32	[0.57; 3.05]	20.4%
Garvey22	10	152	15	152	0.67	[0.31; 1.44]	20.8%
Rubino22	14	253	5	85	0.94	[0.35; 2.53]	19.5%
Jastreboff22	65	1266	2	643	16.51	[4.05; 67.20]	16.8%
Wilding21	65	1306	44	655	0.74	[0.51; 1.07]	22.5%
**Random effects model**		**3080**		**1671**	**1.44**	**[0.48; 4.32]**	**100.0%**
**Prediction interval**						**[0.02; 88.53]**	

Heterogeneity: $I^2 = 79\%$, $\tau^2 = 1.3607$, $p < 0.01$

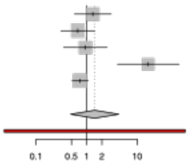

Pancreatitis

Study	Experimental Events	Experimental Total	Control Events	Control Total	RR	95%-CI	Weight
O'Neil18	0	103	1	136	0.44	[0.02; 10.68]	16.2%
Garvey22	0	152	0	152			0.0%
Rubino22	1	253	0	85	1.01	[0.04; 24.61]	16.2%
Jastreboff22	2	1266	1	643	1.02	[0.09; 11.18]	28.7%
Wilding21	3	1306	0	655	3.51	[0.18; 67.89]	18.8%
Pi–Sunyer15	7	2481	0	1242	7.51	[0.43; 131.40]	20.1%
**Random effects model**		**5561**		**2913**	**1.67**	**[0.46; 6.05]**	**100.0%**
**Prediction interval**						**[0.21; 13.47]**	

Heterogeneity: $I^2 = 0\%$, $\tau^2 = 0$, $p = 0.69$

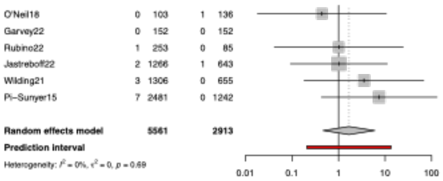

Dyspepsia

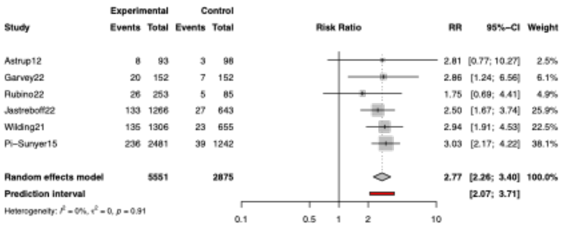

Eructation

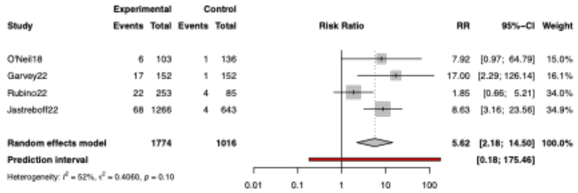

Fatigue

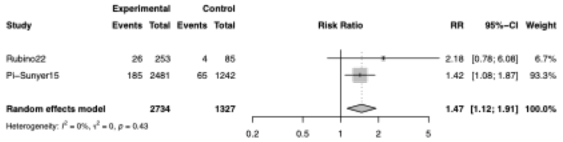

Flatulence

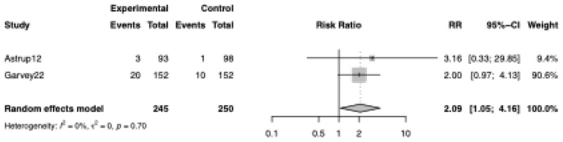

Supplementary Table 1: Probability of net benefit over 1 and 2 years of GLP-1 agonists treatments

**Weight loss target**	**Treatments**	**Year 1**	**Year 1†**	**Year 2**	**Year 2†**
5%	All combined	0.13	(0.26)	0.01	(0.08)
5%	Semaglutide	0.23	(0.39)	0.06	(0.20)
5%	Liraglutide	0.24	(0.39)	0.07	(0.22)
5%	Tirzepatide	0.04	(0.08)	0.01	(0.02)
10%	All combined	0.97	(0.99)	0.91	(0.97)
10%	Semaglutide	0.98	(0.98)	0.96	(0.98)
10%	Liraglutide	0.81	(0.90)	0.72	(0.90)
10%	Tirzepatide	0.79	(0.86)	0.60	(0.77)

† Analysis considering declining events of some harm outcomes over time

Supplementary Figure 3. Contribution of outcomes to the benefit-harm balance of GLP-1 agonists

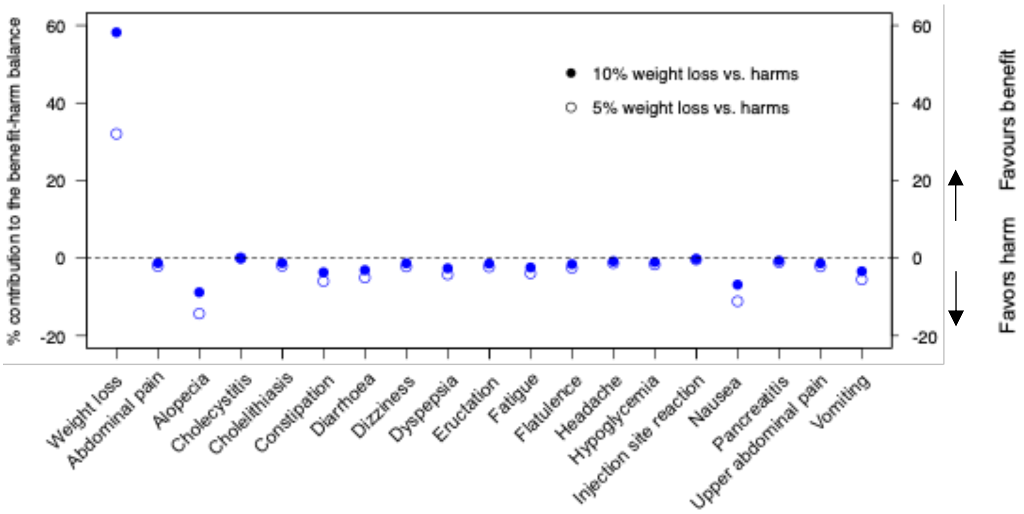

References

1. Yebyo HG, Aschmann HE, Puhan MA. Finding the Balance Between Benefits and Harms When Using Statins for Primary Prevention of Cardiovascular Disease. Ann Intern Med [Internet]. 2018;170(1):1. Available from: http://dx.doi.org/10.7326/m18-1279
2. Yebyo HG, Schmann HE, Menges D, Boyd CM, Puhan MA. Net benefit of statins for primary prevention of cardiovascular disease in people 75 years or older: a benefit–harm balance modeling study. Ther Adv Vaccines. 2018;9(6):259–61.
3. Puhan MA, Yu T, Stegeman I, Varadhan R, Singh S, Boyd CM. Benefit-harm analysis and charts for individualized and preference-sensitive prevention: Example of low dose aspirin for primary prevention of cardiovascular disease and cancer. BMC Med. 2015;13(1):1–11.
4. Yu T, Fain K, Boyd CM, Singh S, Weiss CO, Li T, et al. Benefits and harms of roflumilast in moderate to severe COPD. Thorax. 2014;69(7):616–22.
5. Yebyo HG, Braun J, Menges D, ter Riet G, Sadatsafavi M, Puhan MA. Personalising add-on treatment with inhaled corticosteroids in patients with chronic obstructive pulmonary disease: a benefit–harm modelling study. Lancet Digit Health [Internet]. 2021;7500(1):1–10. Available from: http://dx.doi.org/10.1016/S2589-7500(21)00130-8
6. Gail MH, Costantino JP, Bryant J, Croyle R, Freedman L, Helzlsouer K, et al. Weighing the risks and benefits of tamoxifen treatment for preventing breast cancer. J Natl Cancer Inst. 1999;91:1829–46.
