# Supplementary material for: Corrigendum to “GLP-1 receptor agonists for weight reduction in people living with obesity but without diabetes: a living benefit-harm modelling study” eClinicalMedicine 2024;73:102661
Source: eClinicalMedicine. 2026 Jun 24;97:104022. doi: 10.1016/j.eclinm.2026.104022 (PMC13320438; doi:10.1016/j.eclinm.2026.104022)
Supplement: Supplementary_Appendix [file mmc1.docx]

**Supplementary material**

The meta-analyses were rerun following removal of Wharton et al. (2023), and the affected results are presented in the revised forest plots.

**All GLP-1 RA on 10% weight loss**

**All GLP-1 RA on 5% weight loss**

**Semaglutide on 10% weight loss**

**Semaglutide on 5% weight loss**

**Supplementary Table 1: Probability of net benefit over 1 and 2 years of GLP-1 RA treatment**

The corrected probabilities of net benefit after removal of Wharton et al. (2023) are shown in the "corrected" columns for both the main analysis and the sensitivity analysis assuming declining harm rates over time (indicated by †).

| **Weight loss** | **Treatments** | **Year 1** |  | **Year 1†** |  | **Year 2** |  | **Year 2†** |  |
| --- | --- | --- | --- | --- | --- | --- | --- | --- | --- |
|  |  | **Original** | **Corrected** | **Original** | **Corrected** | **Original** | **Corrected** | **Original** | **Corrected** |
| 5% | All GLP-1 RAs combined | 0.13 | 0.14 | 0.26 | 0.26 | 0.01 | 0.01 | 0.08 | 0.08 |
| 5% | Semaglutide | 0.23 | 0.24 | 0.39 | 0.40 | 0.06 | 0.06 | 0.20 | 0.21 |
| 10% | All GLP-1 RAs combined | 0.97 | 0.96 | 0.97 | 0.98 | 0.91 | 0.88 | 0.97 | 0.96 |
| 10% | Semaglutide, | 0.98 | 0.98 | 0.98 | 0.98 | 0.96 | 0.96 | 0.98 | 0.98 |

† Analysis considering declining events of some harm outcomes over time
